# Letter from Richmond

**Published:** 1861-09

**Authors:** J. G. Westmoreland

**Affiliations:** Richmond, Va.


### ARTICLE II.

Richmond, Va., August 16, 1861.

*Dear Doctor :*

I have been in Virginia only five days, and have therefore had but little chance to make definite reports of the health of the various divisions of the army of the Confederate States in this section.

At this place are found many from Gen. Beauregard's Army prostrate with ordinary diseases, as well as the unfortunate wounded of the Manassas battle. In the General Hospital at this place are found, not only the sick and wounded of our own people, but the wounded of the enemy; for here humanity's call has been heard by our generous and christian people. I visited to-day the Elemosinary Institution where they, with some of our sick soldiers, are cared for. I have seen many of the wounded of both armies with every variety of gunshot injuries. The recuperative energies of the soldiers here are remarkable indeed. Several I observed having serious wounds of vital organs seem to be convalescent, and give strong hopes of perfect recovery. Some cases in which a ball had passed entirely through the lung, are steadily improving, and only one thus injured manifests symptoms which may be considered unfavorable to recovery. The wound of our townsman and friend, Dr. B. M. Smith, is of this character, the ball passing through the right cavity of the chest and around the lung. He is well cared for at a Hotel in this city, and indulges strong hopes of being able in a week or two to return home to Georgia; but will not listen to any suggestion of his friends to remain at home longer than is necessary to attend his duties in the army.* Of two amputations at the hip-joint, one gives probability of recovery—the other case died a few hours after the operation. One shoulder-joint amputation is doing well with good prospect of recovery.

I find those in the Medical Wards of the Hospital, and in Hotels and private houses in the city, laboring under ordinary disease, generally affected with Fever, Measles, Mumps, &c.

The cases of fever are perhaps pretty equally divided between Typhoid and Remittent. While perhaps the same locality would not very likely produce both of these types of fever, yet from the different points in the neighborhood of Manassas, of Winchester, and other places, whence sick soldiers are brought to this place, we should not expect the same variety of disease, as these places differ, doubtless, in many respects, calculated to induce different diseases. No great malignancy seems to attend any of the cases I have seen, and from the length of duration reported for the fatal cases, I am inclined to think the ordinary casualities led to the fatal termination.

---

* We regret to announce the death of our esteemed fellow-citizen, Dr. B. M. Smith, since the above letter was written.—Ed.

While on a visit to my brother, who has charge of the General Hospital of Gen. Magruder's army in the neighborhood of Williamsburg, I had an opportunity of inquiring into the nature of diseases affecting the soldiers in that locality. Measles, though not so prevalent as a few weeks ago, having gone principally through with those liable to attack, is yet to be found, from what I have seen and heard, in every part of our armies. The fatality, so far as I can learn, is not greater than we find in civil practice, notwithstanding the necessity which often exists for crowding large numbers together in hospitals where they are tended. At this season of the year the disease is, in my opinion, as liable to prove fatal in home practice, as in winter, from the great tendency to Diarrhœa, which often leads to a fatal termination. It is reasonable to suppose, however, that in camp life the risk would necessarily be greater in winter on account of the powerful influence that the unavoidable exposure would exert on the respiratory organs, which are perhaps more universally the seat of irritation and inflammation as a consequence of measles. Most of our troops in the field will have obtained an immunity against this disease before the cold season sets in. I find mumps also very common amongst the soldiers I have visited.

A mild form of Diarrhœa prevails in some of the camps I have visited very extremely. In some instances half you meet with in a Regiment are, or have been the subjects of slight derangement of bowels. I am happy to find, however, in my investigations thus far, that no malignant bowel affections exist, either in the form of Diarrhœa or Dysentery.

I noticed in the Hospital, Rheumatism, Pneumonia, Bronchitis, Tuberculosis, &c., but the disease most common was that of fever; and as at this place (Richmond) supplied from

Manassas, &c., the cases are pretty equally divided between Typhoid and Remittent fevers.

The Remittent is of that peculiar type that was so prevalent in the neighborhood of Zebulon in 1847. Irritation of the stomach and bowels, particularly the former, is the particular characteristic. A fiery redness of the edges and top of the tongue; one of the evidences of this is almost constantly present. As this is the symptom thought to be one of the diagnostic signs of Typhoid Fever, more critical examinations are necessary to determine its nature than under other circumstances. Indeed, in this whole region where fever is found amongst the soldiers, I find a disposition on the part of physicians to adopt the expectant plan of treatment, evidently from the want of certainty in regard to its variety in a particular case. I had the satisfaction of testing my pathognononic of Typhoid Fever in the General Hospital at Williamsburg, and also the collection of symptoms proving the existence of Remittent Fever. Would that diagnosis were more studied, and these two forms of fever more readily distinguished from each other, and promptly treated than is too often, unfortunately for suffering humanity—soldiers in particular—found to be the case.

The great difficulty is that the true pathology is not considered, or in many instances ever thought of. Fever is the result of a poison affecting some organ, not all the organs. Malaria affects primarily, the same organ invariably. So with the peculiar cause of Typhoid fever. Now, if this could be believed by all, and the particular organ in each of these fevers thus affected known to all, there would be comparatively little difficulty in diagnosis and correspondingly little in the treatment.

J. G. Westmoreland.

